# Colorimetric and Fluorescent Dual-Modality Sensing Platform Based on Fluorescent Nanozyme

**DOI:** 10.3389/fchem.2021.774486

**Published:** 2021-11-17

**Authors:** Yejian Wan, Jingwen Zhao, Xiaochun Deng, Jie Chen, Fengna Xi, Xiaobo Wang

**Affiliations:** ^1^ Guangxi Medical University Cancer Hospital, Guangxi Medical University, Nanning, China; ^2^ Department of Chemistry, Zhejiang Sci-Tech University, Hangzhou, China

**Keywords:** dual-modality sensing, colorimetric detection, fluorescent detection, nanozyme, graphene quantum dots

## Abstract

Compared with natural enzymes, nanozymes based on carbonaceous nanomaterials are advantages due to high stability, good biocompatibility, and the possibility of multifunctionalities through materials engineering at an atomic level. Herein, we present a sensing platform using a nitrogen-doped graphene quantum dot (NGQD) as a highly efficient fluorescent peroxidase mimic, which enables a colorimetric/fluorescent dual-modality platform for detection of hydrogen peroxide (H_2_O_2_) and biomolecules (ascorbic acid-AA, acid phosphatase-ACP) with high sensitivity. NGQD is synthesized using a simple hydrothermal process, which has advantages of high production yield and potential for large-scale preparation. NGQD with uniform size (3.0 ± 0.6 nm) and a single-layer graphene structure exhibits bright and stable fluorescence. N-doping and ultrasmall size endow NGQD with high peroxidase-mimicking activity with an obviously reduced Michaelis–Menten constant (*K*_m_) in comparison with natural horseradish peroxidase. Taking advantages of both high nanozyme activity and unique fluorescence property of NGQD, a colorimetric and fluorescent dual-modality platform capable of detecting H_2_O_2_ and biomolecules (AA, ACP) with high sensitivity is developed as the proof-of-concept demonstration. Furthermore, the mechanisms underlying the nanozyme activity and biosensing are investigated.

## Introduction

Nanozymes are artificial nanomaterials with enzyme-mimicking properties (Gao et al., 2007; Ju et al., 2016; Sun et al., 2018; Yan, 2020). They promise a wide range of applications (e.g., sensing, catalysis) by overcoming the drawbacks of natural enzymes, including high cost and poor stability (Ding et al., 2019; Xu et al., 2019; Jiao et al., 2020; Liu et al., 2020; Wang et al., 2020; Wang and Wei, 2020; Zhang et al., 2020). In addition, the unique and tunable physicochemical properties of nanomaterials can not only endow nanozymes with multiple functionalities (e.g., optical or magnetic properties), but also provide vast possibilities for rational design for tailored properties (Liu et al., 2019a; Liu et al., 2019b; Jin et al., 2019). In comparison with noble or transition metal–based nanozymes, carbon-based nanozymes are attractive because of their high biocompatibility and chemical stability (Garg et al., 2015; Sun et al., 2015; Garg and Bisht, 2016; Wen et al., 2017; Zeng et al., 2017; Lu et al., 2018; Yang et al., 2020; Li et al., 2022).

Graphene quantum dots (GQDs) or 0D graphene materials, which are atomically thin and nanometer-wide planar carbon structures, are promising for a spectrum of novel applications [e.g., sensing (Bian et al., 2017; Shen et al., 2017; Haque et al., 2018), imaging (Chen et al., 2018; Yan et al., 2019), display (Zhao et al., 2020), anticounterfeiting (Li et al., 2018), catalysis (Li et al., 2015; Tian et al., 2018; Yan et al., 2018; Yan et al., 2020), and energy storage and conversion (Xi et al., 2019)] owing to their molecular size, quantum-confinement-induced bandgap opening, fluorescence, good dispersibility, highly tunable chemicophyscial properties, high chemical and photostability, and good biocompatibility (Wang et al., 2014; Lin et al., 2015). Studies also show that GQDs with functional groups and heteroatom dopants can exhibit nanozyme properties (Ju et al., 2016). However, the current GQD nanozymes are usually synthesized from expensive precursors (e.g., carbon nanotube) using environmentally unfriendly, time-consuming processes (e.g., oxidative cutting in hot concentrated nitric acid).

Sensitive detection of important small molecules or biomolecules using simple and low-cost assays is of great significance in health-related monitoring, diagnosis, and treatment. Hydrogen peroxide (H_2_O_2_) implicates in many biological processes. It is a product of various enzymatic reactions, an important signal molecule, and an indicator of oxidative stress in biological systems (Han et al., 2020). For example, any substrates of oxidoreductases (e.g., glucose, cholesterol, lactate) can be detected because the corresponding enzymatic reactions produce H_2_O_2_. Thus, detection of H_2_O_2_ provides a universal strategy for the detection of a variety of biomarkers and biological states (Wang et al., 2018). Ascorbic acid (AA) is a reducing bioactive molecule, and its antioxidant properties help to prevent cancer development, enhance immunity, and protect cholesterol from oxidative damage. Detection of AA is important because its imbalance in the body is associated with a series of diseases. For macromolecules, acid phosphatase (ACP, EC 3.1.3.2) is a phosphatase ubiquitous in the human body. Abnormally elevated ACP levels indicate prostate or kidney diseases. In comparison with the current detection methods (e.g., electrochemical detection, high-performance liquid chromatography, etc.), optical sensing based on colorimetric and fluorescence detection has the unique advantages of simple and fast operation, high sensitivity, potential of real time, and direct visual monitoring. In contrast to detection using a single readout, a sensing assay based on multisignals is attractive because it simultaneously provides more than one mode of signal output, leading to high diversity and good accuracy. Thus, exploration of a new colorimetric and fluorescence dual-mode sensing platform with simplicity in operation, high sensitivity, and efficiency for detection of small molecules or biomolecules is highly desired.

**FIGURE 1:**
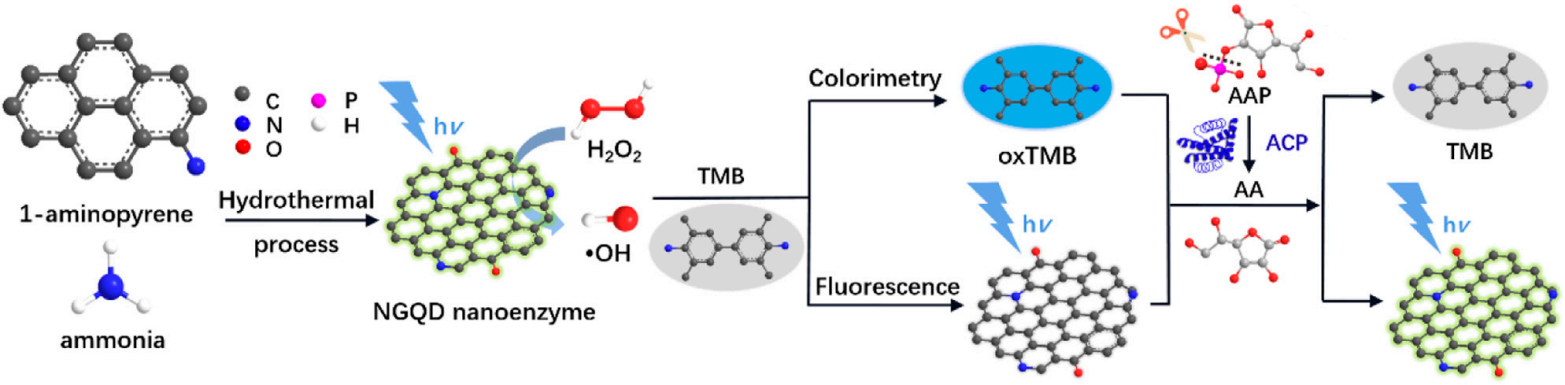
Schematic illustration for the one-step preparation of NGQD nanozymes and the colorimetric/fluorescent dual-modality sensing of H_2_O_2_, AA, and ACP.

In this work, we demonstrate a colorimetric and fluorescent dual-modality platform based on a nitrogen-doped graphene quantum dot (NGQD) fluorescent nanozyme, which is able to detect a spectrum of analytes (Figure 1). In this platform, NGQD, that is, facile and one-step synthesized with gram-scalable production, serves as peroxidase mimics with high activity. In addition to nanozyme-catalyzed colorimetric sensing, the fluorescent property of NGQD also enable simultaneous fluorescent sensing. As the proof-of-concept demonstrations, this technique is employed to detect H_2_O_2_ and biomolecules (AA, ACP) with high sensitivity. In comparison with other nanozymes, the NGQD nanozyme has the advantages of simple and scalable synthesis, high activity, and potential of mass production. The dual-mode sensing based on these multifunctional nanozymes further extend the applications of carbonaceous nanozymes.

## Materials and Methods

### Chemicals and Materials

3,3′, 5,5′-tetramethylbenzidine, 5,5-dimethyl-1-pyrroline-n-oxide, AA, ACP, and L-ascorbic acid-2-phosphate (AAP) were obtained from Sigma-Aldrich (United States). 1-aminopyrene, riboflavin, methionine, nitrotetrazolium chloride blue, terephthalic acid, ethylenediamine tetraacetic acid disodium salt, alanine (Ala), tryptophan (Trp), aspartate (Asp), phenylalanine (Phe), tyrosine (Tyr), threonine (Thr), leucine (Leu), glutamic (Glu), arginine (Arg), histidine (His), ethanol, and ammonia were purchased from Aladdin (China). All chemicals were of analytical grade. Ultrapure water (18.2 MΩ cm) was used to prepare aqueous solution throughout the work.

### Synthesis of NGQD

Using 1-aminopyrene as the precursor and ammonia (0.4 M) as the medium, NGQDs were synthesized hydrothermally. After reaction at 200°C for 6 h, a reddish brown solution is resulted without any solid precipitation. Unreacted molecules were removed through dialysis for 2 days using a dialysis bag with cutoff molecular weight of 1000 Da. The dialysate was filtered through a microporous membrane (0.22 µm) and freeze-dried to obtain NGQD powder. Undoped GQDs were prepared using the same protocol but without adding ammonia.

### Characterization

Transmission electron microscopy (TEM) images were obtained at 200 kV from a transmission electron microscope (JEM-2100; JEOL, Japan). Freshly peeled mica was used as the substrate to deposit NGQDs for atomic force microscopy (AFM) measurement. Tapping mode was employed to obtain AFM images on a Bruker Multimode 8 (Bruker, United States). X-ray photoelectron spectroscopy (XPS) was obtained with Mg Ká radiation (250 W, 14 kV) on an electron spectrometer (PHI5300; Perkin-Elmer, United States). The ultraviolet-visible (UV-Vis) absorption and fluorescence spectra were taken by a UV-Vis spectrometer (UV-2450; Shimadzu, Japan) and a fluorescence spectrometer (RF-5301PC; Shimadzu), respectively. The fluorescence emission spectrum was obtained when excited at 465 nm, and the fluorescence excitation spectrum was measured using an emission wavelength of 520 nm. The absolute photoluminescence (PL) quantum yield was determined by a fluorescence spectrometer (FL 3C-11; Hariba Scientific, United States). Electron paramagnetic resonance (EPR) spectrum was recorded on an EMX-10/12 spectrometer (Bruker, Germany).

### Assays for Nanozyme Activity

The catalyzed reduction of H_2_O_2_ into radicals and the subsequent oxidization of 3,3′,5,5′-tetramethylbenzidine (TMB) was used to determine the peroxidase-like activity of NGQDs (Hu et al., 2018). Specifically, NGQDs (10 μg/ml) were added in the mixture of H_2_O_2_ (6.6 mM) and TMB (0.5 mM) dissolved in HAc-NaAc (0.1 M, pH 4). The UV-vis absorption spectrum and absorbance at 652 nm were obtained after reaction for 10 min. Terephthalic acid (TA, 0.5 mM) was applied to capture •OH radicals upon decomposition of hydrogen peroxide (50 mM) catalyzed by NGQDs (10 μg/ml). The reaction was performed at 37°C for 12 h. Then, the fluorescence spectrum was recorded with an excitation wavelength of 315 nm. For EPR measurement, HAc-NaAc buffer (0.1 M, pH 4.0) containing dimethyl pyridine N-oxide (DMPO) (20 mM) and H_2_O_2_ (20 mM) was applied as the supporting solution. The spectra before and after addition of NGQDs (10 μg/ml) were measured.

The possible oxidase, catalase, or superoxide dismutase (SOD)-mimicking activities of NGQDs were measured according to the literature (Chen et al., 2019a). The activity of oxidase was obtained by measuring the absorbance of TMB solution (0.5 mM in HAc-NaAc buffer, pH = 4.0) after it was directly oxidized by NGQDs (10 μg/ml). The assay of catalase-mimicking activity is based on the decrease of characteristic UV absorption of H_2_O_2_ (10 μM) at 240 nm after its decomposition catalyzed by NGQDs (10 μg/ml). The activity of superoxide dismutase was determined by the improved tetrazolium blue method (Chen et al., 2019a). Briefly, riboflavin was reduced under light conditions, and the reduction product produced •O_2_^−^ in the presence of O_2_, which could further reduce nitrotetrazolium blue (NBT) to blue methylhydrazone with characteristic absorption at 560 nm. Materials with SOD activity can eliminate •O_2_^−^ and inhibit the formation of methylhydrazone. Specially, the absorbance of the mixture solution (in 0.2 M phosphate buffered saline, pH = 7.4) containing riboflavin (85 μM), NBT (1 mM), methionine (5 mM), and EDTA (2.5 mM) at 560 nm with or without NGQDs (10 μg/ml) was measured.

### Detection of H_2_O_2_, AA, and ACP

The mixture of NGQDs (10 μg/ml) and TMB (0.5 mM) in HAc-NaAc buffer (0.1 M, pH 4) was used as the medium. To detect H_2_O_2_, different concentrations of H_2_O_2_ were introduced into the medium at 37°C for 10 min, followed by colorimetric or fluorescence detection. The reduction of oxidated TMB (oxTMB) reports the presence of AA. Specifically, oxTMB was first generated by adding H_2_O_2_ (6.6 mM) in the medium for 30 min reaction at 37°C. Then, different concentrations of AA were added to the oxTMB solution and incubated at 37°C for 10 min, followed by measurement of UV-vis absorption or fluorescence spectrum (excited at 465 nm). The same method was used to determine ACP, for which AA was first generated by preincubating different concentrations of ACP with L-ascorbic acid-2-phosphate (AAP, 20 μM) at 37°C for 30 min (Fan et al., 2018).

## Results and Discussion

### Facile and Scalable Synthesis of NGQDs

A nitrogen (N) atom in the catalytic center of natural enzymes often plays a key role owing to its electron-rich nature (large electronegativity of 3.04 on the Pauling scale) and high catalytic activity toward oxygen reduction or evolution reactions (Fan et al., 2016; Pillar-Little et al., 2018). Thus, an N dopant may endow nanomaterials, such as GQDs, with enzyme-like activity. Wang et al. demonstrates a bottom-up synthesis of GQDs in alkaline solutions using 1,3,6-trinitropyrene as the precursor. However, the synthesis involves nitration of pyrene using hot HNO_3_ (refluxing at 80°C for 12 h) and total removal of N through nucleophilic substitution reactions between NO_2_ groups and alkaline species (e.g., OH groups) (Lin et al., 2015).

**FIGURE 2:**
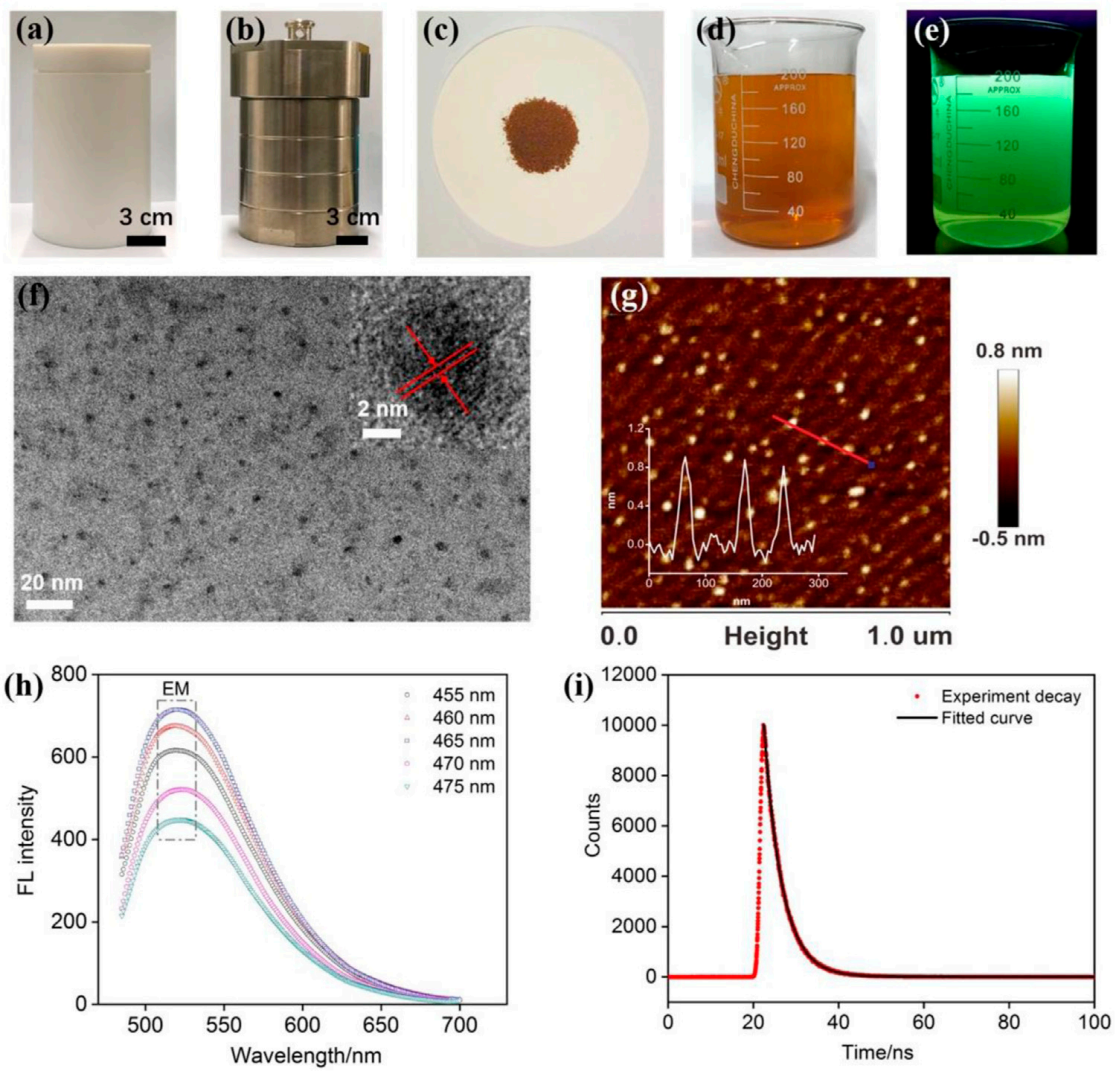
**(A,B)** Photographs of internal Teflon reactor **(A)** and stainless steel housing **(B)** of an autoclave for the preparation of NGQDs. **(C)** Powder of NGQDs obtained by one-pot synthesis. **(D,E)** Photographs of NGQD solution (2 mg/ml) under visible **(D)** or 365 nm UV lights **(E)**. **(F)** TEM image. Insets are HRTEM image **(top)** and size distribution of NGQDs. **(G)** AFM image. Inset shows the height profile along the red line. **(H)** Fluorescence emission spectra obtained at different excitation wavelengths. **(I)** Fluorescence lifetime spectrum.

As illustrated in Figure 1, NGQDs were synthesized in this work through one-step, bottom-up molecular fusion in hydrothermal conditions using 1-aminopyrene as the precursor, which possesses a honeycomb carbon structure like graphene and amino groups. Ammonia solution (NH_4_OH) is employed as the dopant source of nitrogen owing to its high reactivity with the defect sites of GQDs under hydrothermal conditions. To achieve gram-scale synthesis, a large-volume (500 ml; 40% actual usage for pressure safety) autoclave is used (Figure 2A,B), which is much larger than the commonly used reactor for GQDs (50 or 100 ml). Reddish brown powder (0.22 g) was obtained with a production yield of 55.0% after the hydrothermal treatment, purification by dialysis, and freeze-drying (Figure 2C). In comparison with the “top-down” synthesis of GQDs that relies on cutting large graphitized carbon materials (e.g., graphene sheets, carbon nanotubes, or carbon black) using different strategies (e.g., oxidative cutting by strong acids), this “bottom-up” synthesis is green, easy, and of high yield. The as-prepared NGQDs disperse well in water (2 mg/ml) and remain stable for months without precipitation (Figure 2D). NGQDs emit bright green fluorescence under UV irradiation (365 nm, Figure 2E).

### Characterizations of NGQDs

As revealed by TEM (Figure 2F), NGQDs have narrowly distributed sizes with an average diameter of 3.0 ± 0.6 nm (103 samples). The lattice spacing of 0.28 nm can be clearly resolved in high-resolution TEM (HRTEM) images, which corresponds to the [100] facet of graphene. Their thickness is ∼0.8 nm as characterized by AFM (Figure 2G), indicating the single-layered graphene structure. As shown in Figure 2H, the PL emission peaks at 520 nm are independent of excitation wavelength and reach the maximum intensity under 465 nm excitation, suggesting that NGQDs are rather homogeneous in size and surface states. The maximum emission wavelength is 465 nm (Supplementary Figure S1 in SI). The absolute PL quantum yield of NGQDs is as high as 13.5% with a fluorescent lifetime of 4.3 ns (Figure 2I). The undoped GQDs that were prepared using the same protocol but without adding ammonia have an absolute PL quantum yield of 9.8%. Thus, the introduction of ammonia as a nitrogen source leads to the improved fluorescence efficiency of the obtained NGQDs. When NGQDs are continuously irradiated by UV light (365 nm, 40 W) for 3 h, the fluorescence intensity remains at 98.8% of the original intensity, indicating good stability against photobleaching. In addition, NGQDs are stored in an indoor environment for 30 days. The remaining fluorescence intensity is 99.5% of the original intensity, suggesting high long-term storage stability. Even in the presence of high concentrations of salt (NaCl, up to 0.5 M), the fluorescence intensity can still remain at 99.5% of the original intensity. Taken together, NGQDs have high stability.

**FIGURE 3:**
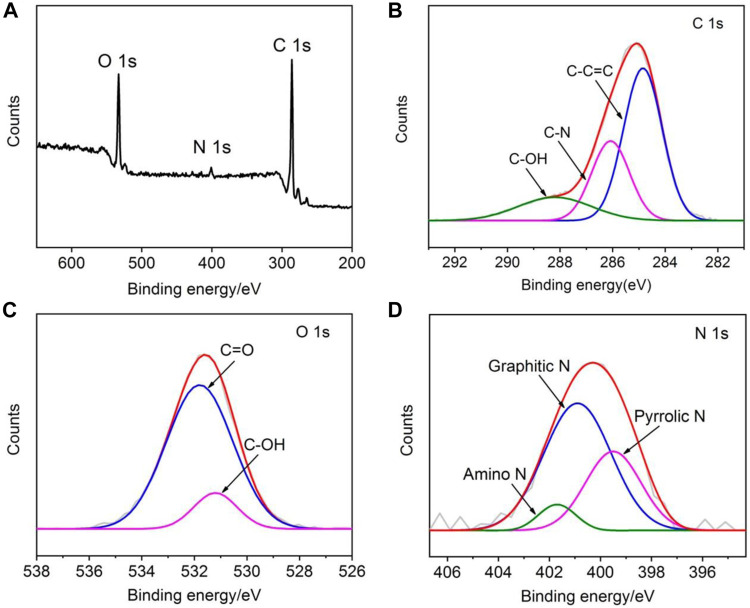
XPS survey spectrum **(A)** and high-resolution C1s **(B)**, O1s **(C)**, and N1s **(D)** spectra of NGQDs.

XPS is used for chemical and elemental analysis of NGQDs. Three characteristic peaks corresponding to C1s, O1s, and N1s are identified in the survey spectrum, revealing the atomic concentrations of C, O, and N in NGQDs of about 76.2%, 20.6%, and 3.2%, respectively (Figure 3A). The peak at a binding energy of 285.9 eV in the high-resolution C1s spectrum confirms the graphitic structure (C-C=C), and the two peaks at 286.3 and 288.5 eV are, respectively, attributed to sp (Ju et al., 2016) C in C-N and C-O bonds, indicating oxygenated and N-containing groups in NGQDs (Figure 3B). The–OH and C=O groups are revealed in the high-resolution O1s spectrum (Figure 3C). The characteristic peaks of amino N, graphitic N, pyrrolic N, and pyridinc N are identified by deconvolving the high-resolution N1s spectrum, confirming that nitrogen is doped in the framework of NGQDs (Figure 3D) (Pillar-Little et al., 2018). Except amino N inherited from the precursor (1-aminopyrene), other N species might be produced through a reaction of NH_4_OH with the defect sites of GQDs under hydrothermal conditions (Tang et al., 2014; Yang et al., 2017).

### The Nanozyme Activity of NGQD and Catalytic Mechanisms

Peroxidase represents a large family of oxidoreductases that catalyze various biological oxidation reactions. Nanozymes with peroxidase-mimicking activities offer a wide range of applications [immunoassays (Zheng et al., 2013), biosensors (Cheng et al., 2019; Zhang et al., 2019), etc.]. Unlike natural enzymes, however, the catalytic activity and specificity of nanozymes are often moderate. Thus, improvement based on nanomaterials engineering is crucial. Heteroatom doping can endow nanomaterials with various new or improved chemico-physical properties. Here, we demonstrate that N-doping confers GQDs with high peroxidase activity.

**FIGURE 4:**
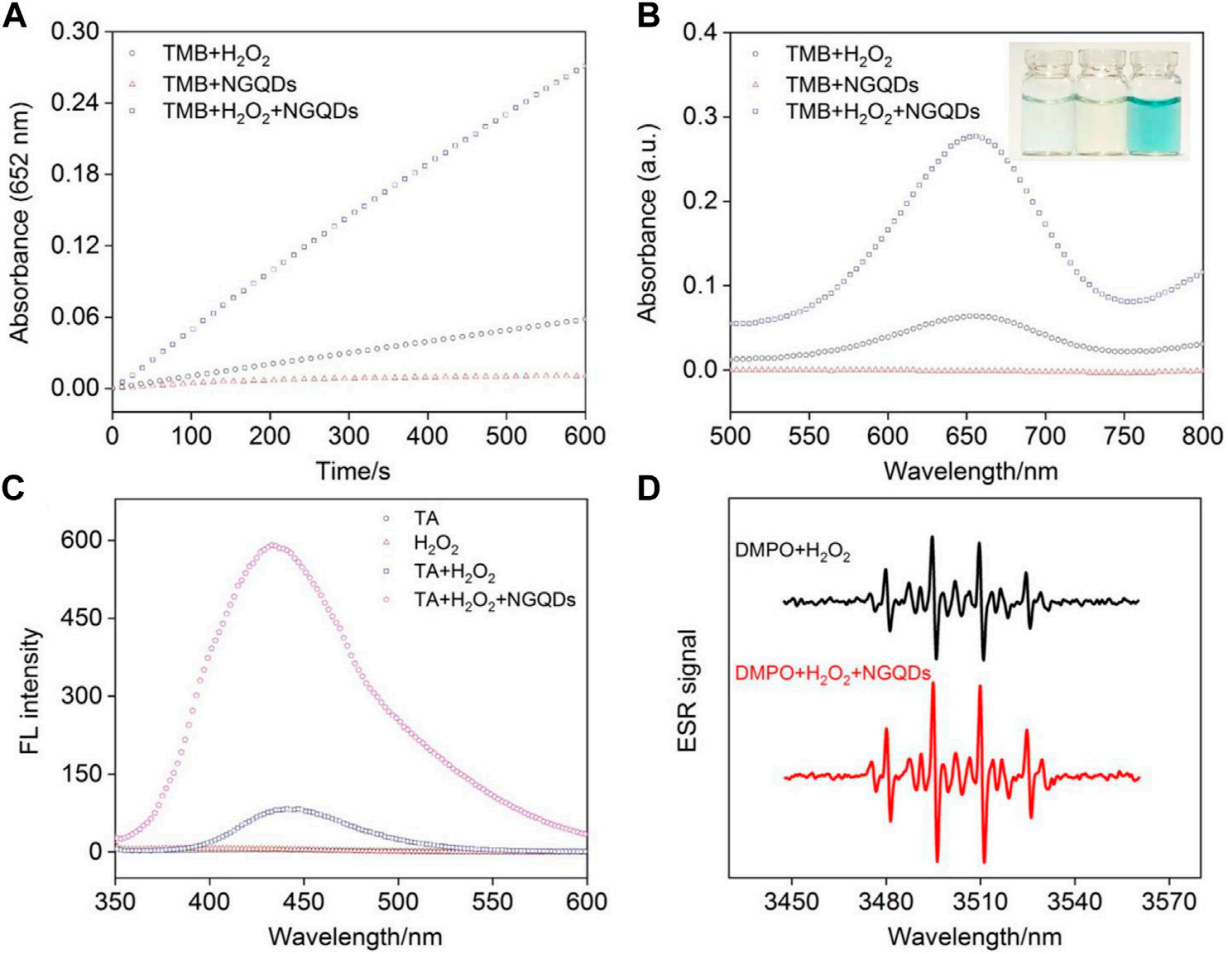
**(A)** Time-dependent change of absorbance at 652 nm and **(B)** absorbance spectra of different mixture solutions of NGQDs, H_2_O_2_, and TMB after 10 min reaction. Inset in b are photographs of TMB solution in the presence of NGQDs **(left)**, H_2_O_2_ **(middle)**, and NGQDs + H_2_O_2_ **(left)**. **(C)** Fluorescence spectra obtained in TA, H_2_O_2_, TA + H_2_O_2_, and TA + H_2_O_2_+NGQDs solutions. **(D)** EPR spectra obtained in the mixture of DMPO and H_2_O_2_ in absence or presence of NGQDs.

The peroxidase activity of NGQDs is reported by the catalyzed reduction of H_2_O_2_ into radicals and subsequent oxidization of TMB into blue colored oxTMB (Figure 1). Based on the change of absorbance at 652 nm determined by a UV-vis spectrometer, this biocatalytic reaction can be monitored in a time-dependent manner. As shown in Figure 4A,B, GQDs alone cannot oxidize TMB. In comparison with the weak reaction in the mixture of H_2_O_2_ and TMB, the ternary system containing NGQDs, H_2_O_2_, and TMB gives an obvious color change, demonstrating the intrinsic peroxidase-like activity of NGQDs. In contrast, undoped GQDs that were synthesized under the same conditions but without the addition of NH_4_OH only show very low peroxidase-mimicking activity (Supplementary Figure S2 in SI). Under the same conditions, the absorbance at 652 nm of the undoped GQD system (ternary solution containing GQDs, H_2_O_2_, and TMB) is only about 20% of that of the NGQD system. Thus, N doping shall be responsible for the improved nanozyme activity of NGQDs. The peroxidase-mimicking activity of NGQDs was also measured when NGQDs (0.2 mg/ml) were stored at pH 4 (0.1 M HAc-NaAc) or at room temperature or with a high concentration of salt (NaCl, 0.5 M) for 7 days. The obtained three NGQDs were then applied to react with H_2_O_2_ and TMB. The absorbance of the mixture at 652 nm is 97.0%, 99.1%, and 98.9% of that obtained using the original NGQDs, indicating negligible changes in nanozyme activity. These phenomena might be ascribed to the high stability of NGQDs. In comparison with natural bioenzymes that commonly need to be refrigerated under neutral pH, NGQD nanozymes exhibit good tolerance to harsh environments.

TA and DMPO were applied as indicators for the generated hydroxyl radical (•OH). The mixture containing TA, H_2_O_2_, and NGQDs exhibited high fluorescence intensity, demonstrating that •OH was produced from the catalytic reaction (Figure 4C). EPR spectra obtained in the presence of DMPO also confirms the production of •OH (Figure 4D) (Song et al., 2010). We speculate that the mechanism for the generation of •OH radicals is due to the presence of C=O groups and C/N heterostructures by N doping. The C=O groups act as the catalytic active centers, and heterostructures improve the electron transfer process, facilitating the formation of •OH radicals through cleavage of O-O bond of H_2_O_2_ (Sun et al., 2015).

We speculate that N dopants in NGQDs can selectively activate H_2_O_2_ by trapping the oxygen atoms of H_2_O_2_ to promote the formation of oxygen radicals, which subsequently oxidize TMB. This similarly explains the peroxidase-mimicking activity of previously reported N-doped carbon nanoparticles (Fan et al., 2018), N-doped reduced graphene oxide (rGO), or mesoporous carbon (Zheng et al., 2013). This catalytic mechanism is also consistent with that for natural enzymes, that is, the iron in the catalytic active center of heme in natural horseradish peroxidase (HRP) promotes the adsorption of O atoms on H_2_O_2_ (Fan et al., 2018). The possibility that NGQD may also mimic other enzymes similar to peroxidase, including oxidase (direct oxidation of TMB by NGQD), catalase (production of O_2_ from NGQD-catalyzed decomposition of H_2_O_2_), and SOD (elimination of •O_2_^−^ by NGQD) were also investigated. As shown (Supplementary Figure S3 in SI), NGQD exhibits negligible oxidase- and SOD-mimicking activities and very low catalase-mimicking activity, indicating that the NGQD nanozyme is highly specific to peroxidase-mimicking activity.

As with other nanozymes or natural enzymes, the peroxidase-like activity of NGQDs is also pH- and temperature-dependent (Supplementary Figure S4 in SI). Similar to a natural peroxidase, the activity of NGQDs maximizes at pH 4, but NGQD exhibits higher thermal stability than natural enzymes. Specifically, NGQD retains 65% of its activity at 50°C compared to 42% for HRP (Sun et al., 2018). In comparison with other representative carbon-based nanozymes, NGQD exhibits higher peroxidase-like activity at a low concentration (10 μg/ml) under similar experimental conditions (Wang et al., 2011; Hu et al., 2018; Yadav et al., 2018). The high enzymatic activity is ascribed to the unique set of merits, including catalytically active N dopants, molecular size, and high dispersibility.

**FIGURE 5:**
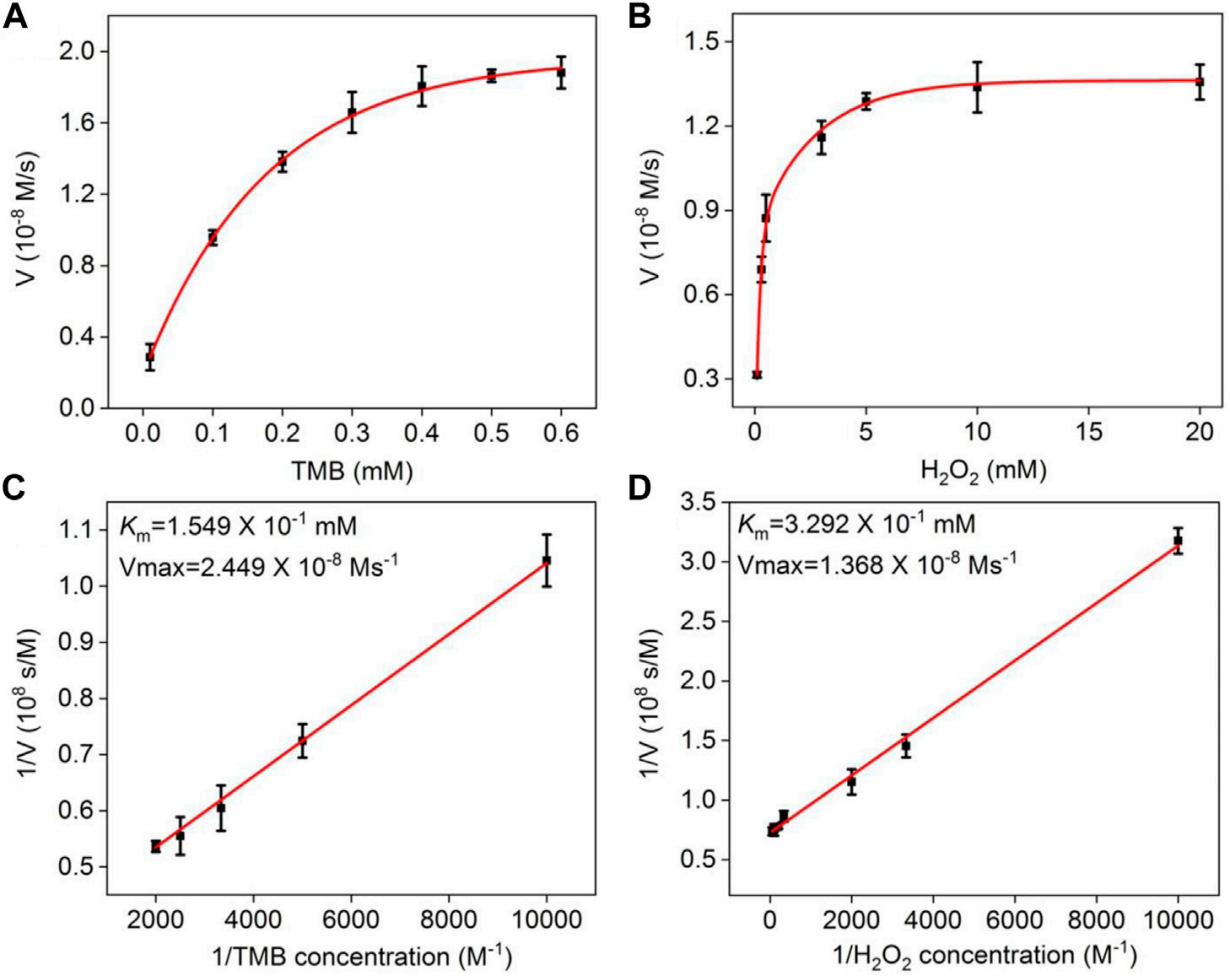
**(A,B)** Steady-state kinetic assay of NGQDs, in which the reaction velocity is determined through oxidation of TMB based on absorption at 652 nm with varying concentrations of **(A)** TMB or **(B)** H_2_O_2_. **(C,D)** Double-reciprocal plots of NGQD activity obtained using Michaelis–Menten model at a fixed concentration of H_2_O_2_ (c, 6.6 mM) or TMB (d, 0.5 mM) versus various concentrations of TMB **(C)** or H_2_O_2_ **(D)**.

The Michaelis–Menten model was employed to analyze the kinetic parameters of the NGQD nanozyme (Hu et al., 2018). As shown in Figure 5, the Michaelis–Menten constant (*K*_m_) and maximum initial velocity (*V*_max_) are obtained from a Lineweaver–Burk plot. The former reflects the binding affinity between the enzyme and substrate, and the latter reveals the maximum rate achieved at the saturating substrate concentration. Using TMB as the substrate, *K*_m_ and *V*_max_ of NGQD nanozyme are 0.1549 mM and 2.449 × 10^−8^ M/s, respectively (Figure 5A,B). The *K*_m_ value is the lowest as compared with that of natural HRP and other carbon-based nanozymes (Li et al., 2016; Bano et al., 2018; Singh et al., 2018; Sun et al., 2018; Hu et al., 2018; Chandra et al., 2019). Using H_2_O_2_ as the substrate, *K*_m_ and *V*_max_ of the NGQD nanozyme are 0.3292 mM and 1.380 × 10^−8^ M/s, respectively (Figure 5C,D). The *K*_m_ value is an order of magnitude lower than the natural enzyme and is lower than that of carboxylated graphene oxide (COOH-GO) (Wang et al., 2011), carbon nanoparticles (Wang et al., 2018), and N-doped carbon dots (N-CDs) (Bano et al., 2018). The obviously reduced *K*_m_ is attributable to the abundant N-dopants on ultrasmall GQDs, which act as the binding sites for H_2_O_2_.

### Colorimetric and Fluorescent Detection of H_2_O_2_

**FIGURE 6:**
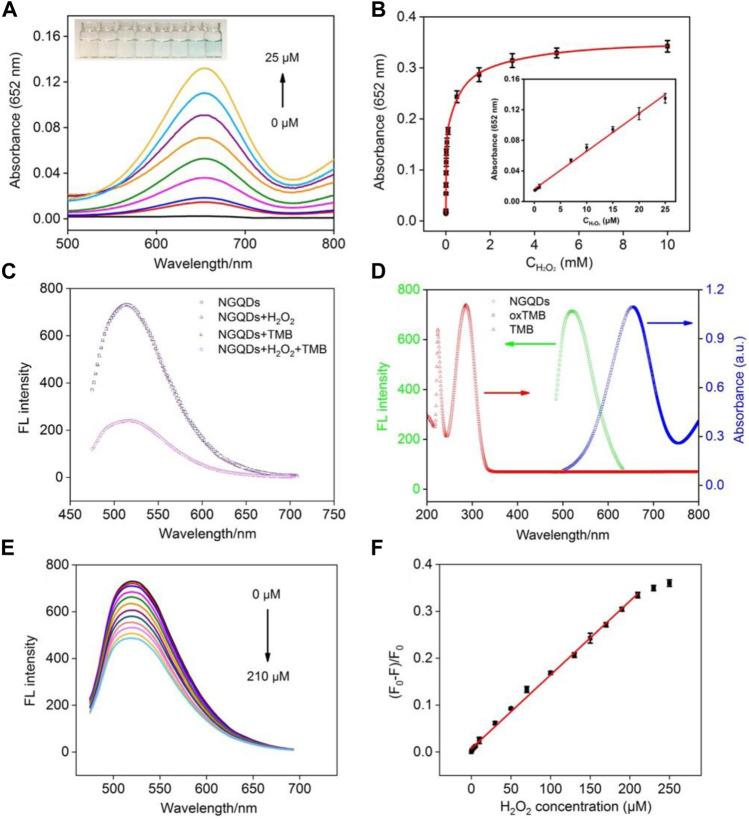
**(A)** Absorbance spectra and photographs (inset) obtained from the mixture of NGQDs and TMB in the presence of different concentrations of H_2_O_2_. **(B)** Change in absorbance at 652 nm with the increasing H_2_O_2_ concentration. Inset is the linear calibration plot for colorimetric detection of H_2_O_2_. **(C)** The fluorescence spectra of NGQDs in the absence or presence of H_2_O_2_, TMB, H_2_O_2_, or their combination. **(D)** Fluorescence spectrum of NGQDs and absorbance spectra of TMB or oxTMB. **(E)** Fluorescence spectra of NGQDs in TMB solution containing different concentrations of H_2_O_2_ (0–210 μM). **(F)** The linear calibration plot for fluorescent detection of H_2_O_2_.

In the presence of NGQDs and TMB, the absorbance of oxTMB at 652 nm increases with increasing concentration of H_2_O_2_ along with the change from colorless to blue (Figure 6A). Good linearity is obtained from this colorimetric detection in the concentration range of 0.1–25 μM with a limit of detection (LOD) of 60 nM at a signal-to-noise ratio (S/N) of 3 (Figure 6B).

Owing to the highly tunable fluorescence properties and high photostability, GQDs show great potential in fluorescence-based sensing. Here, we for the first time combine the nanozyme and fluorescence property for biosensing. As shown in Figure 6C, TMB does not quench the fluorescence of NGQDs. In the presence of H_2_O_2_, the fluorescence of NGQDs also remains unchanged despite the generation of hydroxyl radicals, whereas the fluorescence of NGQDs is significantly quenched while having both TMB and H_2_O_2_. Thus, the fluorescence quenching of NGQDs is caused by oxTMB. The lifetime of NGQDs in the presence of oxTMB and H_2_O_2_ remain the same (4.3 ns), suggesting static quenching without electron transfer (Supplementary Figure S5 in SI) (Chen et al., 2018; Yan et al., 2019). In addition, as revealed by TEM, florescence quenching is not caused by aggregation of NGQDs (Supplementary Figure S6 in SI). As shown in Figure 6D, the fluorescence spectra of NGQD and the absorption spectrum of oxTMB overlap in the wavelength range of 500–700 nm. Thus, the fluorescent emission from NGQDs can be adsorbed by oxTMB, leading to fluorescence quenching.

Taken together, it is conceivable that a sensing platform based on the fluorescence and nanozyme properties may be constructed. Figure 6E shows detection of H_2_O_2_ based on fluorescence quenching using NGQDs as both peroxidase-mimicking nanozyme and fluorescent reporters. Good linear correlation is found between the ratio of fluorescence quenching and the concentration of H_2_O_2_ from 0.5 to 210 μΜ (Figure 6F). The LOD is 120 nM at a S/N of 3. As demonstrated, detection of H_2_O_2_ can be realized using both colorimetric and fluorescent methods based on NGQDs.

### Dual-Modality Detection of AA and ACP

The sensitive response of NGQDs toward H_2_O_2_ provides a universal strategy to detect a variety of molecules. In addition, molecules that can decrease the concentration of H_2_O_2_ or react with oxTMB can also be detected. When AA (50 μM) is added in the solution containing NGQDs, TMB and H_2_O_2_, the blue color from the produced oxTMB gradually fades away because AA reduces oxTMB back to TMB (inset in Supplementary Figure S7 in SI). AA can be sensitively detected using both colorimetric (Supplementary Figure S7 in SI, linear range of 10∼90 μM with LOD of 4.1 μM) and fluorescence (Supplementary Figure S8 in SI, linear range of 5∼70 μM with LOD of 3.6 μM) modes. The selectivity for AA detection is investigated by testing the fluorescence quenching ratio obtained in the mixture of NGQDs and TMB in presence of uric acid (UA), dopamine (DA), different types of amino acids, or reducing agents (Supplementary Figure S9 in SI). As seen, UA and DA that usually coexist with AA and significantly interfere with the determination of AA in electrochemical sensing have a negligible effect on AA detection. The tested amino acids other than cysteine (Cys) also have no significant interference with the detection. When Cys, glutathione (GSH), or homocysteine (Hcy) with reducibility are tested, reduced fluorescence of NGQDs can be found, indicating the reaction with oxTMB. However, AA results in the highest reduction of fluorescence, suggesting the highest activity. The influence of coexisting reducing substances can be eliminated by establishing a standard curve for detection using the sample matrix as the supporting medium. On the other hand, the accurate concentration of AA can also be obtained using linear extrapolation in a standard recovery method.

**FIGURE 7:**
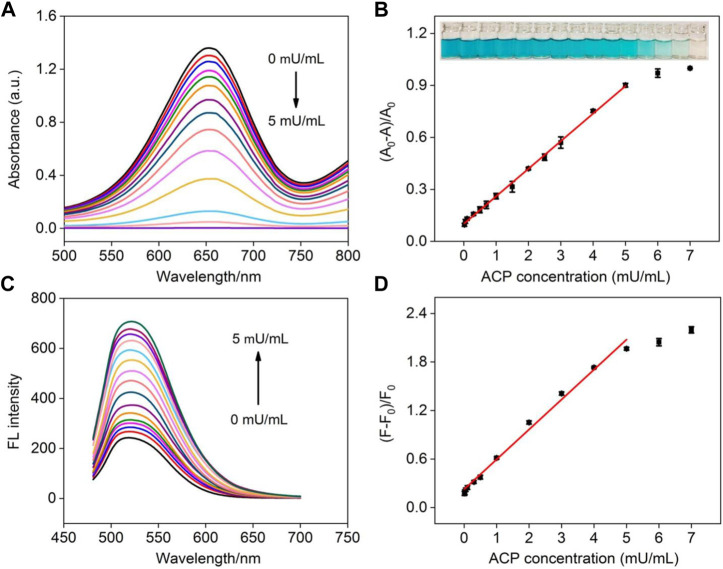
**(A)** Absorbance spectra obtained in the mixture of NGQDs + H_2_O_2_ + TMB in the presence of different concentrations of ACP (0–5 mU/ml). **(B)** Linear calibration plot for colorimetric detection of ACP. Insets are photographs of the corresponding solutions (from low to high concentrations of ACP). **(C)** The fluorescence spectra of NGQDs obtained in the mixture of NGQDs + H_2_O_2_ + TMB in the presence of different concentrations of ACP (0–5 mU/ml). **(D)** The linear calibration plot for ACP detection in fluorescent detection.

As illustrated in Figure 1, AA is the specific hydrolysis product of AAP in the present of ACP. Therefore, ACP can also be detected by the NGQD nanozyme in the presence of TMB and H_2_O_2_ after being incubated with AAP to produce AA. As depicted in Figure 7A, the blue solution colored by oxTMB gradually fades away with the increase of ACP concentration. Good linearity is obtained using colorimetric detection in the concentration range of 20∼5 mU/ml with an LOD of 14 μU/ml (S/N = 3) (Figure 7B). Comparison between determination of ACP using different electrodes is demonstrated in Supplementary Table S1 (SI) (Hu et al., 2016; Deng et al., 2017; Chen et al., 2019b; Lin et al., 2020; Zhang et al., 2021). The LOD is lower than that obtained from palladium square nanoplates on reduced graphene oxide (PdSP@rGO) (Chen et al., 2019b), chitosan modified platinum nanoparticles (Ch-PtNPs) (Deng et al., 2017), and acridone derivative 10-benzyl-2-amino-acridone (Zhang et al., 2021). The started concentration in the detection linear range is lower than that obtained using bathocuproinedisulfonate complex and molybdenum oxide nanoparticles (MoO_3_ NPs) (Hu et al., 2016; Lin et al., 2020). For the detection using a fluorescence signal channel, a linear detection range of 10∼5 mU/ml with an LOD of 4.6 μU/ml (S/N = 3) is obtained. The LOD is lower than that obtained using N-CDs, N-CDs-MnO_2_ nanocomposites, or Eu^3+^-coordination polymer (Supplementary Table S1 in SI) (Zhu et al., 2018; Zhu et al., 2019; Li et al., 2021). To investigate the specificity of ACP detection, the detection system was, respectively, treated with ACP, bovine serum albumin, trypsin, glucose oxidase, pepsin, or lysozyme. As shown in Supplementary Figure S10 (SI), the fluorescence signal dramatically changed in the presence of ACP, and the other enzymes or proteins exhibit negligible effects, indicating high specificity of detection.

The practicability and reliability of the developed dual-modality detection are assessed by detecting ACP in serum (diluted by a factor of 10). As shown in Supplementary Table S2 (SI), the recoveries of colorimetric determination of ACP range from 98.9% to 106.6% and the relative standard deviation values are no more than 3.8%. For fluorescent detection (Supplementary Table S3 in SI), satisfactory recoveries between 100.8% and 104.2% are obtained. In addition, the results obtained using colorimetric and fluorescent determination is close, indicating high accuracy of the dual-modality detection. Compared with the commonly used ACP detection methods (e.g., electrochemistry, colorimetry, fluorescence, potentiometric immunoassay, surface-enhanced Raman spectroscopy, and chromatography), our nanozyme-based detection is simple, convenient, fast, and sensitive.

## Conclusion

In summary, we develop a colorimetric/fluorescence dual-modality sensing platform based on the NGQD nanozyme. NGQD is synthesized using a one-step, bottom-up method in aqueous solution, which is simple, green, of low-cost, and easily scalable. The obtained NGQD exhibits high peroxidase-mimicking activity as well as a bright and stable fluorescence property. Such a novel fluorescent nanozyme may be employed for various applications, such as sensing, photo-catalysis, chemical synthesis, antimicrobial agents, and flexible devices. In comparison with other nanozymes, our NGQD is synthesized by a high-yield, convenient, one-pot, scalable, and low-cost method, and it is catalytically efficient and selective. As the proof-of-concept demonstration, NGQD is utilized here for a colorimetric/fluorescence dual-modality sensing platform that can be used to sensitively detect a variety of chemicals, biomolecules, and physiological states. In comparison with other nanozymes, our NGQD possesses the advantage of convenient and low-cost synthesis and high catalytical efficiency. Owing to highly tunable chemico-physical properties through materials engineering at an atomic level, the multifunctional GQD nanozyme, therefore, allows vast opportunities for dual-mode sensing in combination with diverse nanozyme substrates.

## Data Availability

The original contributions presented in the study are included in the article/Supplementary Material, further inquiries can be directed to the corresponding authors.
