# Supplementary material for: Colorimetric and Fluorescent Dual-Modality Sensing Platform Based on Fluorescent Nanozyme
Source: Front Chem. 2021 Nov 17;9:774486. doi: 10.3389/fchem.2021.774486 (PMC8635524; doi:10.3389/fchem.2021.774486)
Supplement: Supplementary file 1 [file Table1.DOCX]

SUPPORTING INFORMATION

Tables

Table S1 Comparison between detection of ACP using different methods.

| **Material** | **Methods** | | **Linear range**  **(mU/mL)** | **Detection limit**  **(μU/mL)** | **Ref.** |
| --- | --- | --- | --- | --- | --- |
| PdSP@rGO | Colorimetric | | 0.5-100 | 92.5 | 55 |
| Cu(BCDS)_2_^2-^ | Colorimetric | | 0-220 | 7.8 | 56 |
| MoO_3_ NPs | Colorimetric | | 0.09–7.3 | 11 | 57 |
| Ch-PtNPs | Colorimetric | | 0.25-2.5 | 16 | 58 |
| BAA | Colorimetric | | 0.05-2.5 | 41.5 | 59 |
| N-CDs-MnO_2_ nanocomposites | Fluorescent | | 5-40 | 100 | 60 |
| N-CDs | Fluorescent | | 1-50 | 430 | 61 |
| Eu^3+^-coordination polymer | Fluorescent | 0.13-5 | | 40 | 62 |
| NGQDs | Colorimetric  Fluorescent | 0.02-5  0.01-5 | | 14  4.6 | Our work |

PdSP@rGO, palladium square nanoplates on reduced graphene oxide; Cu(BCDS)_2_^2-^, bathocuproinedisulfonate complex; MoO_3_ NPs, molybdenum oxide nanoparticles; Ch-PtNPs, chitosan modified platinum nanoparticles; BAA, acridone derivative 10-benzyl-2-amino-acridone; N-CDs, N-doped carbon dots.

**Table S2** **Colorimetric determination of ACP in serum samples.**

| Sample | Added  (mU/mL) | Found  (mU/mL) | RSD  (%) | Recovery  (%) |
| --- | --- | --- | --- | --- |
| Serum^a^ | 0.5 | 0.5057 | 3.6 | 101.1 |
|  | 1 | 1.066 | 1.9 | 106.6 |
|  | 3 | 2.968 | 3.8 | 98.9 |

^a^ diluted by a factor of 10.

**Table S3 Fluorescent determination of ACP in serum samples.**

| Sample | Added mU/mL | Found  mU/mL | RSD  (%) | Recovery  (%) |
| --- | --- | --- | --- | --- |
| Serum^a^ | 0.5 | 0.5212 | 2.7 | 104.2 |
|  | 1 | 1.012 | 1.4 | 101.2 |
|  | 3 | 3.024 | 1.9 | 100.8 |

^a^ diluted by a factor of 10.

Figures

**
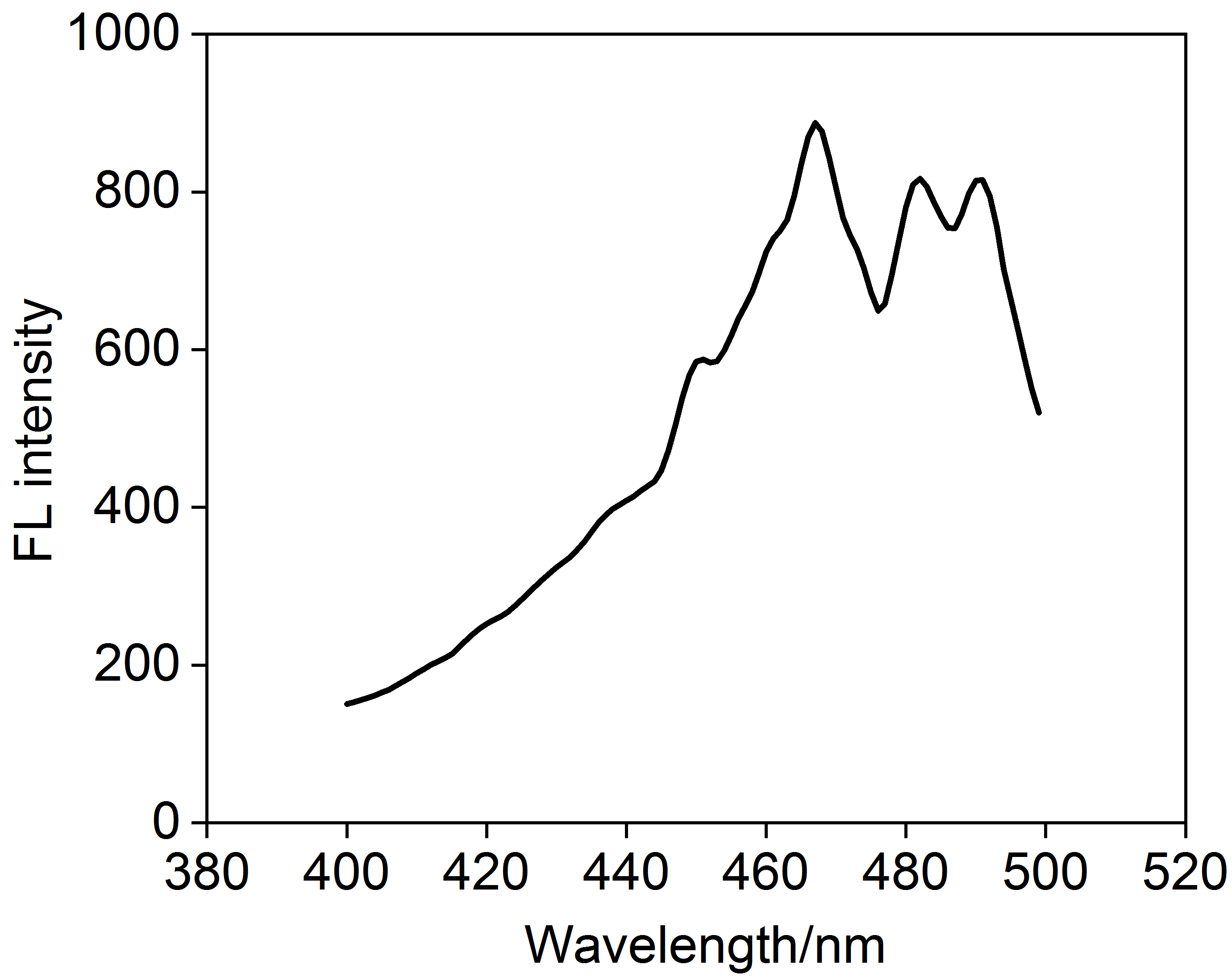
**

**Figure S1** Fluorescence excitation spectrum of NGQD.

**
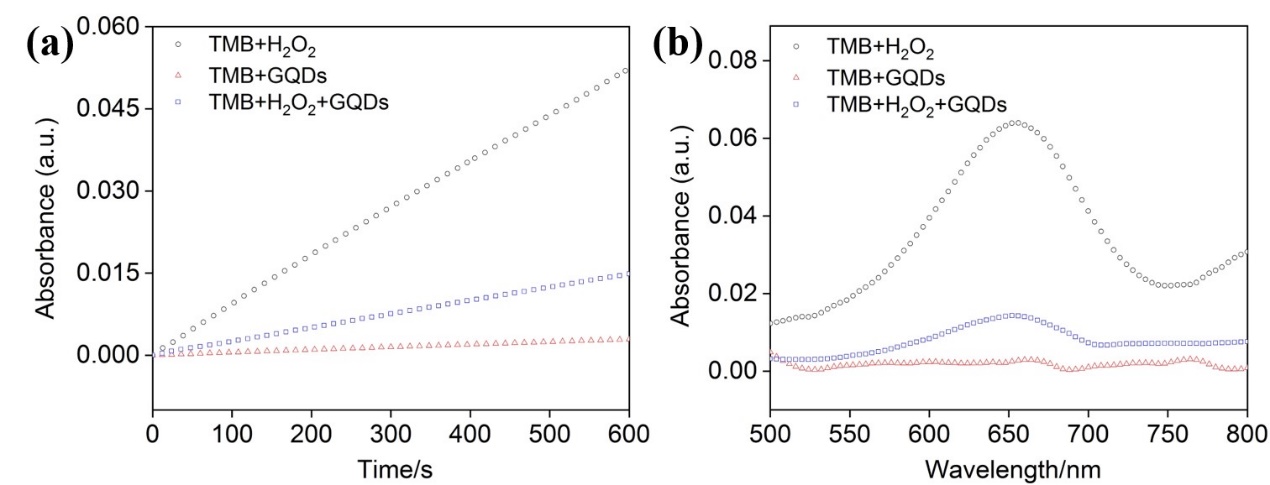
**

**Figure S2** (a) Time-dependent absorbance changes and (b) absorbance spectra at 652 nm in mixture of undoped GQDs, H_2_O_2_ and TMB after reacted for 10 min.


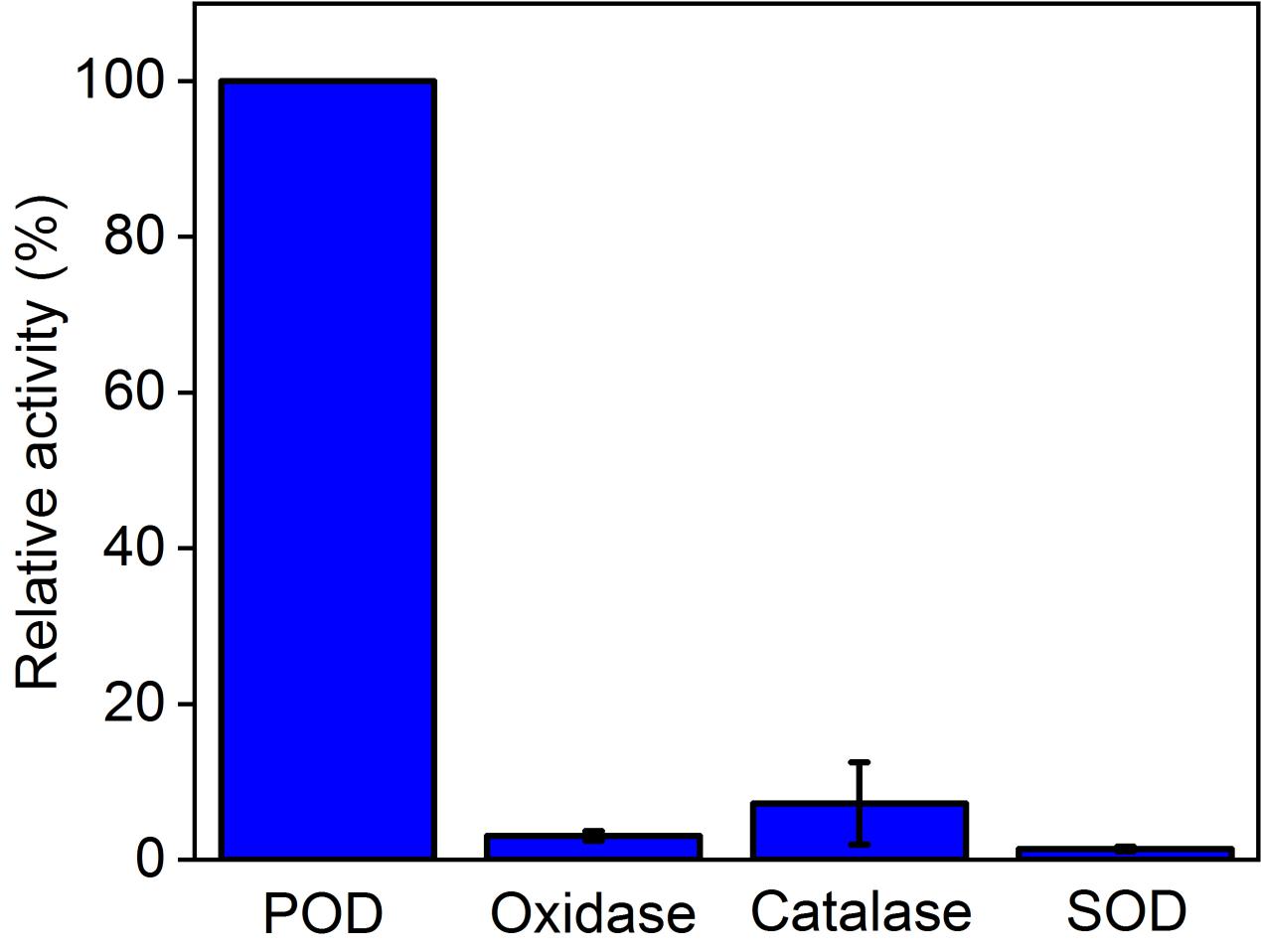


**Figure S3** Peroxidase-, oxidase-, catalase- and SOD-mimicking activities of NGQDs.


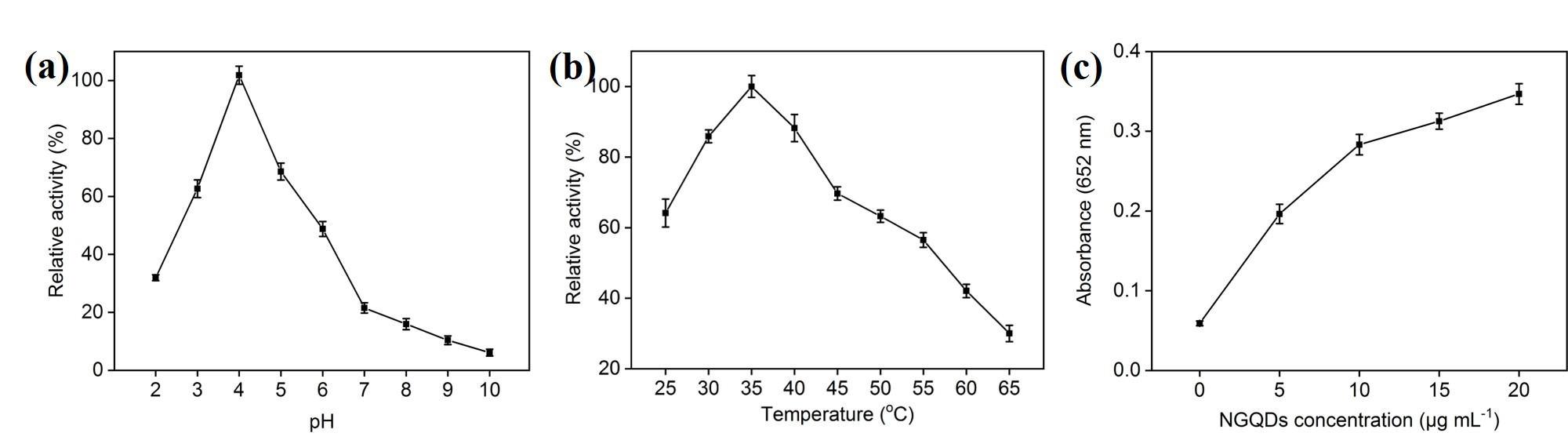


**Figure S4** The dependence of peroxidase-like activity of NGQDs on (a) pH, (b) temperature and (c) the concentration of NGQDs.


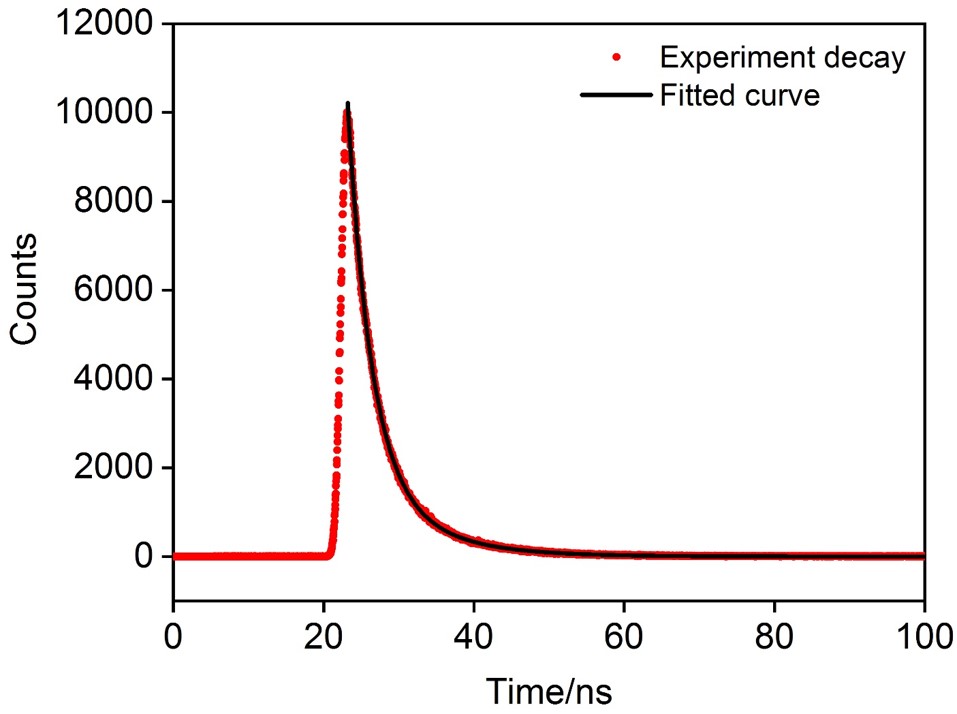


**Figure S5** The FL lifetime spectrum of NGQDs in presence of H_2_O_2_ and TMB.





**Figure S6** TEM image of NGQDs in presence of H_2_O_2_ and TMB.


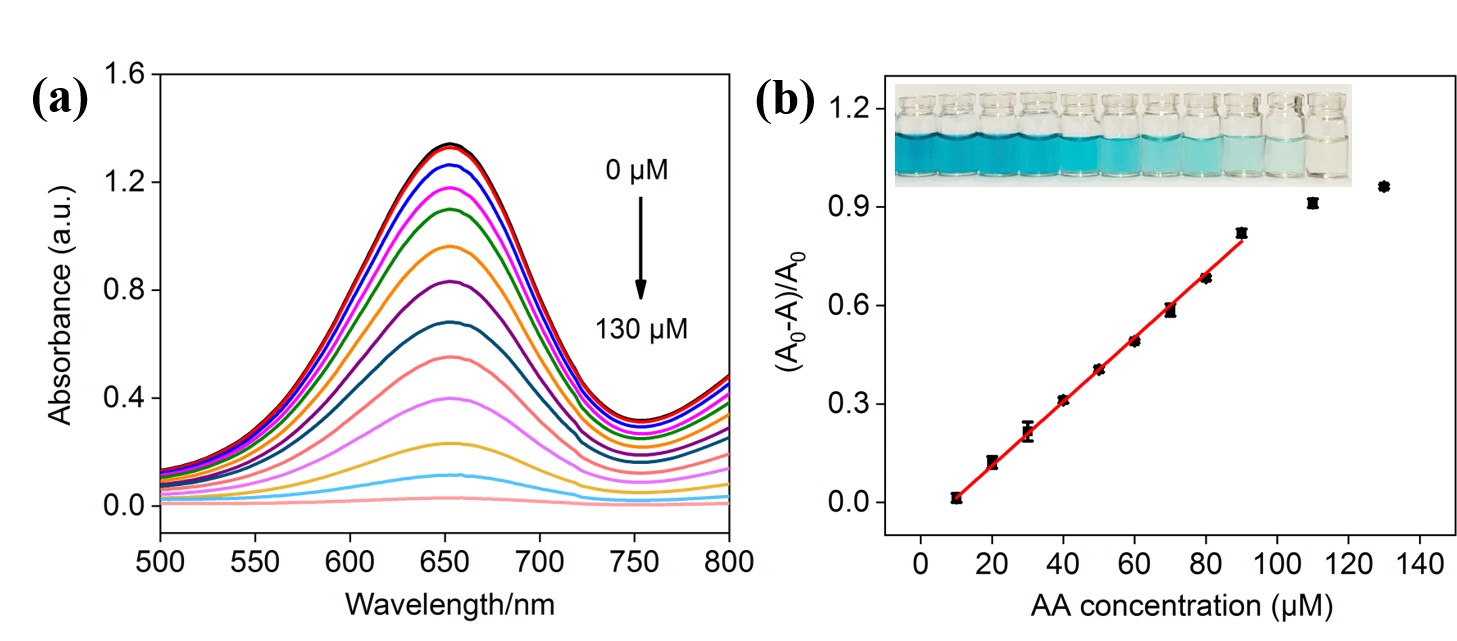


**Figure S7** (a) Absorbance spectra obtained in the mixture of NGQDs and TMB in the presence of different concentrations of AA. (b) The linear calibration plot for AA detection using colorimetric mode. Insets are photographs of the corresponding solutions.


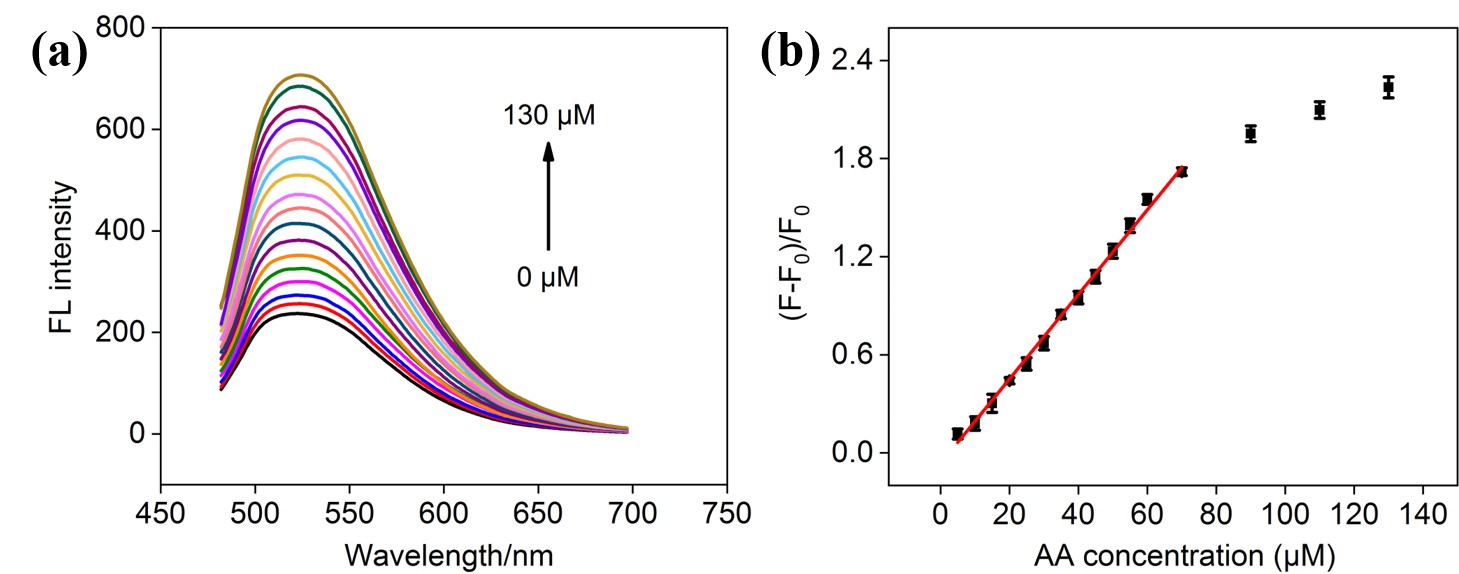


**Figure S8** (a) Fluorescence spectra obtained in the mixture of NGQDs and TMB in the presence of different concentrations of AA. (b) The linear calibration plot for AA detection using fluorescent mode.


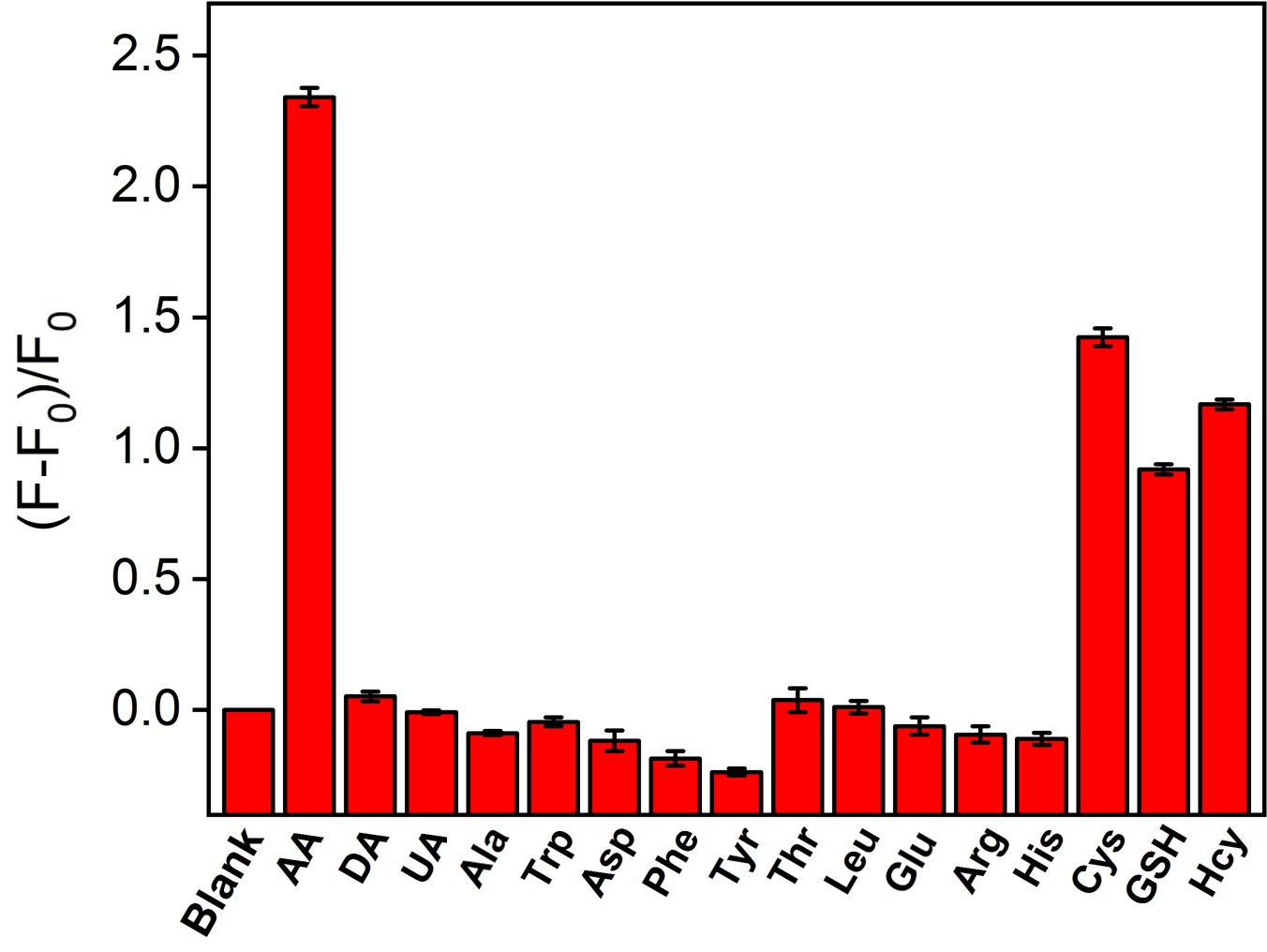


**Figure S9** Fluorescence quenching ratio obtained in the mixture containing NGQDs (10 μg/mL), TMB (0.5 mM) and H_2_O_2_ (6.6 mM) in the presence of different molecules (130 μM). F_0_ and F are fluorescence intensity in absence or presence of the indicated molecules, respectively.


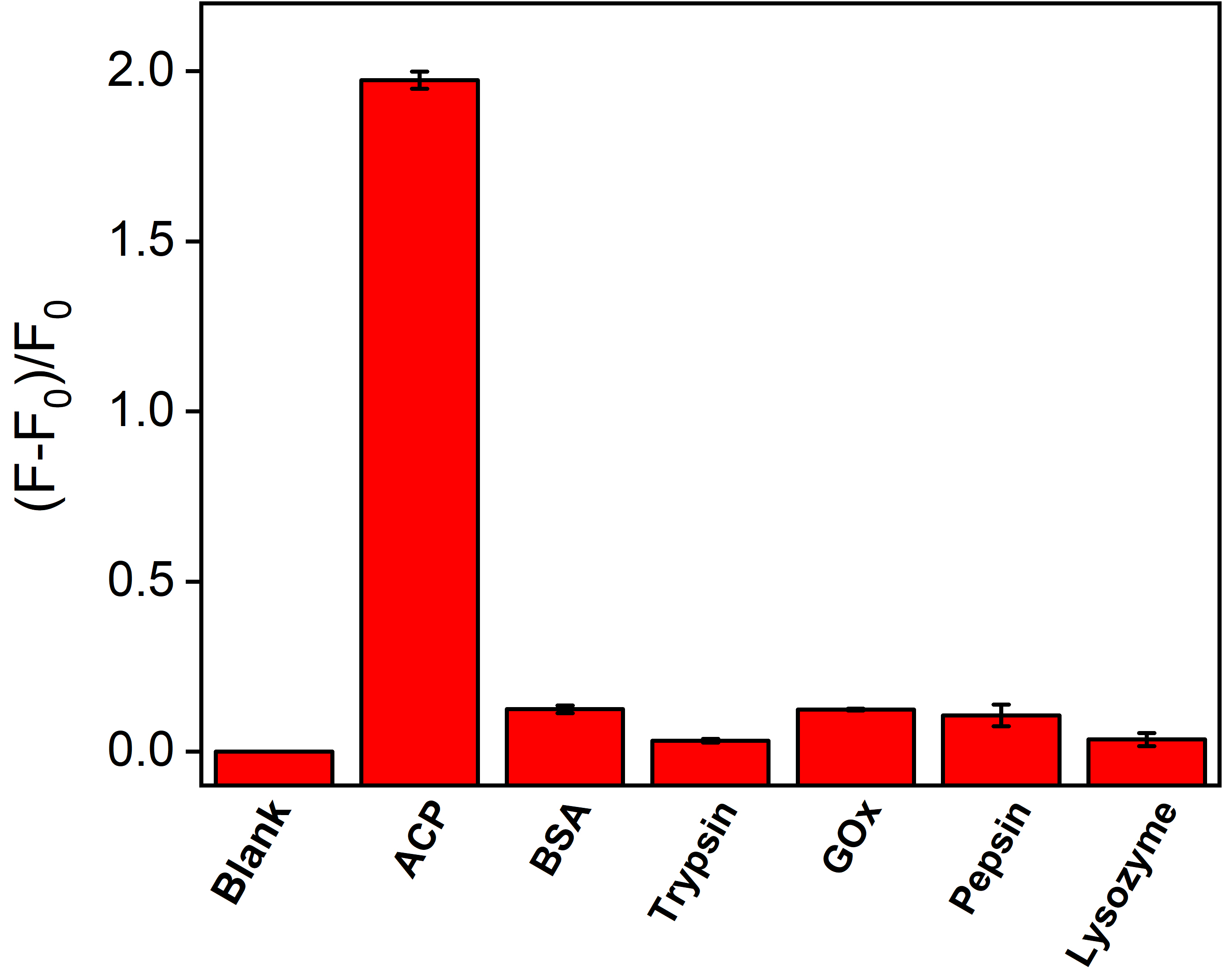


**Figure S10** Fluorescence quenching ratio obtained in the mixture containing AAP (20μM), TMB (0.5 mM) and H_2_O_2_ (6.6 mM) in the presence of ACP (3 mU/mL) or other enzyme or protein (20 ug/mL). F_0_ and F are fluorescence intensity in absence or presence of the indicated biomolecules, respectively.
